# Abnormal calcium homeostasis in heart failure with preserved ejection fraction is related to both reduced contractile function and incomplete relaxation: an electromechanically detailed biophysical modeling study

**DOI:** 10.3389/fphys.2015.00078

**Published:** 2015-03-20

**Authors:** Ismail Adeniran, David H. MacIver, Jules C. Hancox, Henggui Zhang

**Affiliations:** ^1^Biological Physics Group, School of Physics and Astronomy, The University of ManchesterManchester, UK; ^2^Department of Cardiology, Taunton and Somerset HospitalMusgrove Park, Taunton, UK; ^3^School of Physiology and Pharmacology and Cardiovascular Research Laboratories, School of Medical SciencesBristol, UK; ^4^School of Computer Science and Technology, Harbin Institute of TechnologyHarbin, China

**Keywords:** heart failure, calcium, 3D model, ventricle

## Abstract

Heart failure with preserved ejection fraction (HFpEF) accounts for about 50% of heart failure cases. It has features of incomplete relaxation and increased stiffness of the left ventricle. Studies from clinical electrophysiology and animal experiments have found that HFpEF is associated with impaired calcium homeostasis, ion channel remodeling and concentric left ventricle hypertrophy (LVH). However, it is still unclear how the abnormal calcium homeostasis, ion channel and structural remodeling affect the electro-mechanical dynamics of the ventricles. In this study we have developed multiscale models of the human left ventricle from single cells to the 3D organ, which take into consideration HFpEF-induced changes in calcium handling, ion channel remodeling and concentric LVH. Our simulation results suggest that at the cellular level, HFpEF reduces the systolic calcium level resulting in a reduced systolic contractile force, but elevates the diastolic calcium level resulting in an abnormal residual diastolic force. In our simulations, these abnormal electro-mechanical features of the ventricular cells became more pronounced with the increase of the heart rate. However, at the 3D organ level, the ejection fraction of the left ventricle was maintained due to the concentric LVH. The simulation results of this study mirror clinically observed features of HFpEF and provide new insights toward the understanding of the cellular bases of impaired cardiac electromechanical functions in heart failure.

## Introduction

Heart failure (HF) is often categorized into two major types (Borlaug and Paulus, 2010; MacIver, 2010a; Phan et al., 2012; Liu et al., 2013; Zouein et al., 2013): HF with reduced ejection fraction (HFrEF) and HF with preserved ejection fraction (HFpEF). The main distinguishing criterion is an arbitrary cut-off value for the left ventricular ejection fraction of >50% for HFpEF (Vasan and Levy, 2000; Zile et al., 2001; Yturralde and Gaasch, 2005; Asrar Ul Haq et al., 2014) and ≤ 50% for HFrEF (Borlaug and Paulus, 2010; Phan et al., 2012; Liu et al., 2013; Zouein et al., 2013). HFpEF and HFrEF are also commonly referred to as diastolic and systolic HF respectively and also share a variety of abnormalities (Borlaug and Paulus, 2010; Soma, 2011; MacIver and Dayer, 2012; Asrar Ul Haq et al., 2014). Currently, the prevalence of HFpEF is ~50% (Konstantinou et al., 2013; Liu et al., 2013; Asrar Ul Haq et al., 2014), but it is predicted to become the dominant form of HF within the next decade (Liu et al., 2013; Asrar Ul Haq et al., 2014).

The leading cause of HFpEF is hypertension and the dominant pathophysiological mechanism is thought to involve impaired relaxation of the left ventricle (LV) (Liu et al., 2013; Asrar Ul Haq et al., 2014). Other distinguishing features cited are increased LV stiffness and elevated LV end-diastolic pressures (Liu et al., 2013; Asrar Ul Haq et al., 2014). In addition, abnormalities of systolic shortening are common with reduced global longitudinal strain, strain rate, reduced midwall fractional shortening, reduced systolic annular motion, increased isovolumetric contraction time and reduced systolic longitudinal shortening velocities in HFpEF (Sanderson, 2007; Wang et al., 2008; Kono et al., 2009; MacIver, 2010a). HFpEF is also characterized by dyspnoea, fluid retention, exercise intolerance, coronary artery disease and atrial fibrillation (Redfield et al., 2003; Bhatia et al., 2006; Owan et al., 2006; Konstantinou et al., 2013; Liu et al., 2013). It has a greater prevalence in older people and females (Liu et al., 2013). Patients also exhibit other comorbidities such as diabetes, obesity, peptic ulcer disease, cancer and chronic obstructive pulmonary disease (Liu et al., 2013).

As relaxation is an active energy-consuming process and dependent on intracellular Ca^2+^ homeostasis (Robertson et al., 1982; Ebashi, 1984; Barry and Bridge, 1993; Bers, 2001; Konstantinou et al., 2013), impaired relaxation in HFpEF may reflect abnormal intracellular Ca^2+^ homeostasis. Indeed, experimental data from clinical electrophysiology and animal model studies have shown that HFpEF is associated with abnormal Ca^2+^ handling, including an increased SR Ca^2+^ leak current (*I_leak_*) and a decreased Ca^2+^ release from ryanodine receptors (Borbély et al., 2005; Selby et al., 2011; Zile and Gaasch, 2011; Trenor et al., 2012; Gomez et al., 2014). In addition, some ionic currents responsible for generating cardiac action potentials are also remodeled, including those carried by the late-sodium channel, the transient outward K^+^ channel, the inward rectifier K^+^ channel, the Na^+^/K^+^ pump (*I_NaK_*), the background Ca^2+^ channel and the Na^+^/Ca^2+^ exchanger (Borbély et al., 2005; Selby et al., 2011; Zile and Gaasch, 2011; Gomez et al., 2014; Trenor et al., 2012). Structurally, HFpEF is also associated with concentric LV hypertrophy with high LV mass/volume ratio, cardiomyocyte hypertrophy and interstitial fibrosis. This is different to HFrEF, where structural remodeling is characterized by progressive ventricular dilation, eccentric LV remodeling, low LV mass/volume ratio, cardiomyocyte loss along with replacement fibrosis (Borlaug and Paulus, 2010; Konstantinou et al., 2013; Asrar Ul Haq et al., 2014).

The functional impacts of HFpEF-related abnormal Ca^2+^ homeostasis, ion channel and structural remodeling on cardiac electro-mechanics are unclear. Thus, the present study was conducted in order: (1) to develop a novel HFpEF electromechanical single cell model based on extant experimental data; (2) to investigate the cellular mechanisms influencing myocardial calcium homeostasis in HFpEF using an electromechanical single cell model; and (3) to evaluate the functional impacts of impaired Ca^2+^ handling, ion channel remodeling and degrees of concentric LV hypertrophy on the electro-mechanical activity of the heart.

## Materials and methods

### Electromechanical single cell model

For electrophysiology (EP), we utilized the O'Hara-Rudy (ORd) human ventricular single cell model (O'Hara et al., 2011), which was developed from undiseased human ventricular data and recapitulates human ventricular cell electrical and membrane channel properties, as well as the transmural heterogeneity of ventricular action potential (AP) across the ventricular wall (O'Hara et al., 2011). The ORd model also reproduces Ca^2+^ vs. voltage-dependent inactivation of L-type Ca^2+^ current and Ca^2+^/calmodulin-dependent protein kinase II (CaMK) modulated rate dependence of Ca^2+^ cycling. For simulating cellular mechanics properties, we used the Tran et al. myofilament (MM) model (Tran et al., 2010). This model was chosen as it is an extension of the well-established cross-bridge cycling model of cardiac muscle contraction model of Rice et al. (2008). In addition to its ability to replicate a wide range of experimental data including steady-state force-sarcomere length (F-SL), force-calcium and sarcomere length-calcium relationships (Rice et al., 2008; Tran et al., 2010), it also reproduces many of the observed effects of MgATP, MgADP, Pi, and H^+^ on force development.

The intracellular calcium concentration from the EP model was used as the coupling link to the MM model. [*Ca*^2+^]_*i*_ produced as dynamic output from the EP model during the AP served as input to the MM model from which the amount of Ca^2+^ bound to troponin is calculated. The formulation of the myoplasmic Ca^2+^ concentration in the human ventricular myocyte electromechanical cell model is:

(1) $$\begin{array}{l} \frac{d{\left[Ca^{2+}\right]}_{i}}{dt}=\beta_{Cai}\cdot (-(I_{pCa}+I_{Cab}-2\cdot I_{NaCa,i})\cdot \frac{A_{cap}}{2\cdot F\cdot v_{myo}}\\ \;-J_{up}\cdot \frac{v_{nsr}}{v_{myo}}+J_{diff,Ca}\cdot \frac{v_{ss}}{v_{myo}}-\frac{J_{Trop}}{1000}) \end{array}$$

where *β_Cai_* is the buffer factor for [*Ca*^2+^]_*i*_, *I_pCa_* (μA/μF) is the sarcolemmal Ca^2+^ pump current, *I_Cab_* (μA/μF) is the Ca^2+^ background current, *I_NaCa,i_* (μA/μF) is the myoplasmic component of Na^+^/Ca^2+^ exchange current, *A_cap_* (cm^2^) is capacitive area, *F* (coul/mol) is the Faraday constant, *v_myo_* (μL) is the volume of the myoplasmic compartment, *v_nsr_* (μL)is the volume of the network sarcoplasmic reticulum compartment, *v_ss_* (μL) is the volume of the subspace compartment, *J_up_* (mM/ms) is the total Ca^2+^ uptake flux, via SERCA pump from myoplasm to the network sarcoplasmic reticulum, *J_diff,Ca_* (mM/ms) is the flux of the diffusion of Ca^2+^ from the subspace to the myoplasm and *J_Trop_* (μM/ms) is the flux of Ca^2+^ binding to troponin calculated via the MM model. β_Cai_ is formulated as:

(2) $$\beta_{Cai}=\frac{1}{1+\frac{[CMDN]\cdot K_{m,CMDN}}{{\left(K_{m,CMDN}+{\left[Ca^{2+}\right]}_{i}\right)}^{2}}}$$

where [CMDN] is the calmodulin Ca^2+^ buffer in the myoplasm and *K_m,CMDN_* is the half-saturation concentration of calmodulin.

### HFpEF electromechanical single cell model

To develop the human electromechanical single cell model of a HFpEF cardiomyocyte, we first modified parameters of the ORd model based on HF experimental data following the work of Gomez et al. (2014), Trenor et al. (2012) on human HF. This resulted in a generic human HF model. We then made alterations based on the cellular and molecular properties of HFpEF (Zile and Gaasch, 2011) to obtain a biophysically detailed model of HFpEF. For the myofilament changes relative to control, we made modifications based on experimental data on HFpEF patients following the work of Borbély et al. (2005), Selby et al. (2011). Table 1 summarizes the extensive modifications to the control ORd cardiomyocyte to produce the HFpEF cardiomyocyte model.

**Table 1:** **Changes in original ORd model to simulate HFpEF and HFrEF**.

**Parameter modified**	**% change in the HFrEF model compared to the normal model**	**% change in the HFpEF model compared to the normal model**
I_NaL_	180%	180%
τ_hL_	180%	180%
I_to_	40%	40%
I_K1_	68%	68%
I_NaK_	70%	70%
I_Nab_	100%	100%
I_NCX_	175%	70%
I_leak_	130%	130%
CaMKa	150%	150%
J_rel,NP,∞_	80%	80%
	**Myofilament (Borbély et al., 2005; Zile and Gaasch, 2011)**	
PCon_titin	x1.00	x2.00
PCon_collagen	x2.36	x2.00
P_Exp_collagen	x0.42	x0.50

*The modified parameters are: the late Na^+^ current (I_NaL_), the time constant of inactivation of the I_NaL_ (t_hL_), the transient outward K^+^ current (I_to_), the inward rectifier K^+^ current (I_K1_), the Na^+^/K^+^ pump current (I_NaK_), the background Ca^2+^ current (I_Cab_), the Na^+^/Ca^2+^ exchanger (I_NCX_), the SR Ca^2+^ leak current (I_leak_), the Ca^2+^/calmodulin-dependent protein kinase II (CaMK), the nonphosphorylated Ca^2+^ release, via ryanodine receptors (J_rel,NP,∞_), passive force factor due to titin (PCon_titin), passive force (F_passive) and active force (F_active). Based on Borbély et al. (2005), Zile and Gaasch (2011), Trenor et al. (2012) and Gomez et al. (2014)*.

#### Parameter sensitivity analysis for influential cellular processes on relaxation in HFpEF

To our knowledge, only the work by Zile and Gaasch (2011) provides any data on the differences in the cellular and molecular processes that influence Ca^2+^ homeostasis in patients with systolic and diastolic HF. We based our HFpEF model on their work (Zile and Gaasch, 2011) as given in section HFpEF Electromechanical Single Cell Model. In simulations, the only difference between the HFrEF and HFpEF models is a reduction in NCX in the HFpEF condition (see Table 1) as compared to the HFrEF condition. In order to determine whether a reduction in NCX is responsible for the poor end-diastolic relaxation in HFpEF, we performed a parameter-dependent sensitivity analysis of the HFpEF model to NCX, by varying NCX from 70% (HFpEF) to 175% (HFrEF).

### Protocols

The pacing protocols used to evaluate Ca^2+^ homeostasis were as follows:

#### Post-rest contractions

We used a post-rest contraction (PRC) protocol to evaluate sarcoplasmic reticulum (SR) Ca^2+^ content, retention, release, reuptake, and leak. After pacing the single cell for 10 min at 1 Hz to allow steady-conditions to be attained, resting intervals of 1, 2, 3, 5, and 10 s were introduced. The resting periods were then followed by a single stimulus. The varying developed indices such as active tension are a reflection of SR Ca^2+^ release.

### Tissue mechanics model

We modeled cardiac tissue mechanics within the theoretical framework of nonlinear elasticity (Marsden and Hughes, 1994; Holzapfel, 2000) as an inhomogeneous, anisotropic, nearly incompressible nonlinear material similar to previous studies (Costa et al., 2001; Whiteley et al., 2007; Niederer and Smith, 2008; Pathmanathan and Whiteley, 2009; Adeniran et al., 2013a,b). We used a two-field variational principle with the deformation *u* and the hydrostatic pressure *p* as the two fields (Bonet and Wood, 2008; Adeniran et al., 2013a,b; Le Tallec). *p* is utilized as the Lagrange multiplier to enforce the near incompressibility constraint. Thus, the total potential energy function Π for the mechanics problem is formulated as:

(3) $$\Pi (u,p)=\Pi_{\text{int}}(u,p)+\Pi_{ext}(u)$$

where Π_int_ (*u*, *p*) is the internal potential energy or total strain energy of the body and Π_*ext*_ (*u*) is the external potential energy or potential energy of the external loading of the body. As in previous studies (Niederer and Smith, 2008; Keldermann et al., 2009; Pathmanathan and Whiteley, 2009; Adeniran et al., 2013a,b), in the absence of body forces, and assuming that the body is always in instantaneous equilibrium and no inertia effects, the coordinates of the deformed body satisfies the steady-state equilibrium equation with near incompressibility enforced.

The values that minimize the total potential energy in Equation (3) are obtained by searching for its critical points in suitable admissible displacement and pressure spaces $\hat{U}$ and $\hat{P}$. The corresponding Euler-Lagrange equations resulting from Equation (3) lead to solving the problem (Braess and Ming, 2005; Auricchio et al., 2010; Boffi et al., 2013; Le Tallec):

Find (*u*, *p*) in $\hat{U}$ × $\hat{P}$ such that:

(4) $$\begin{array}{l} \int_{\Omega }\frac{\partial \hat{W}}{dF}(x,Id+\nabla u):\nabla vdx-\int_{\Omega }p\frac{\partial \text{det}}{dF}(x,Id+\nabla u):\nabla vdx\\ \;=\int_{\partial \Omega }g.vds\;\forall v,u\in \hat{U}\\ \int_{\Omega }q\left[\text{det}(Id+\nabla u)-1\right]dx=0\;\forall v,u\in \hat{P} \end{array}$$

where $\hat{U}$and $\hat{P}$ are the admissible variation spaces for the displacements and the pressures, respectively. *F* = *Id* + ∇*u* is the deformation gradient, *v* is a test function and $\hat{W}$ is the material stored energy function and corresponds to the density of elastic energy locally stored in the body during the deformation.

The deformation gradient *F* is a tensor that maps elements from the undeformed configuration to the deformed configuration (Marsden and Hughes, 1994; Holzapfel, 2000). Following Cherubini et al. (2008), Ambrosi et al. (2011), we multiplicatively decompose *F* into a microscopic (active) component and a macroscopic elastic (passive) component:

(5) $$F=F_{e}F_{0}$$

The active component *F_o_* measures the length change of the tissue due to muscle contraction while the passive component *F_e_* accounts for the passive mechanical response of the tissue and possible tension due to external loads.

With the vector fields *f, s, and n* denoting the unique direction of the fibers, sheets and sheet-normals in the undeformed state of the LV, the microscopic active component of the deformation tensor *F* takes the form:

(6) $$F_{o}=I+\gamma_{f}f⊗f+\gamma_{s}s⊗s+\gamma n⊗n$$

where *I* is the identity tensor and γ*_x_* is a scalar field that represents the intensity of the contraction, i.e., the active strain in the appropriate direction. γ_*f*_ is defined as:

(7) $$\gamma_{f}=\frac{SL-SL_{0}}{SL_{0}}$$

where *SL* is the new sarcomeric length and *SL*_0_ is the resting sarcomere length of the electromechanical single cell. Following the work of (Rossi et al., 2014)

$$\gamma_{n}=K\gamma_{f},\gamma_{s}=\frac{1}{\left(1+\gamma_{f})(1+\gamma_{n}\right)}-1$$

Thus, γ > 0 denotes elongation, and γ < 0 denotes contraction. The parameter *K* is the link between the microscopic and the macroscopic active deformations (Bogaert and Rademakers, 2001; Rossi et al., 2014) and in our simulations, we used *K* = 4, according to experimental observations (Rademakers et al., 1994; Rossi et al., 2014).

The elastic component *F_e_* is formulated as:

(8) $$F_{e}=FF_{0}^{\;-1}$$

and the corresponding Right Cauchy-Green strain tensor is:

(9) $$C_{e}=F_{e}^{T}F_{e}$$

The associated Green-Lagrange strain tensor is:

(10) $$E_{e}=\frac{1}{2}(C_{e}-I)$$

To characterize the constitutive behavior of cardiac tissue, we used a mixed formulation of the compressible neo-Hookean strain energy function *W* (Auricchio et al., 2010):

(11) $$\hat{W}=\frac{\mu }{2}[I:\hat{C}-d]-\mu \text{ln}\hat{J}+p\text{ln}\hat{J}-\frac{p^{2}}{2\lambda }$$

where μ and λ are positive constants, “:” represents the usual inner product for second-order tensors, $\hat{C}$ is the right Cauchy-Green deformation tensor, *d* is the spatial dimension and $\hat{C}$ = det$\hat{J}$ is the Jacobian of the deformation gradient.

### Tissue electrophysiology model

The monodomain representation (Colli Franzone et al., 2005; Keener and Sneyd, 2008; Adeniran et al., 2013a,b) of cardiac tissue was used for the electrophysiology model:

(12) $$C_{m}\frac{dv}{dt}=-(I_{ion}+I_{stim})+\nabla \cdot (D\nabla V)$$

where *C_m_* is the cell capacitance per unit surface area, *V* is the membrane potential, *I_ion_* is the sum of all transmembrane ionic currents from the electromechanics single cell model, *I_stim_* is an externally applied stimulus and *D* is the anisotropic diffusion tensor given by:

(13) $$D=\sigma_{f}(f⊗f)+\sigma_{s}(s⊗s)+\sigma_{n}(n⊗n)$$

σ*_f_*, σ*_f_*, and σ*_f_* are the intracellular conductivities in the fiber, sheet and cross-sheet directions respectively. These were set to 3.0, 0.1, and 0.31525 ms·mm^−1^ respectively. These gave a conduction velocity of 65 cm·s^−1^ in the fiber direction along multiple cells, which is close to the value 70 cm·s^−1^ observed in the fiber direction in human myocardium (Taggart et al., 2000). To guard against any drift in the steady state values of the ion concentrations in the model, the electromechanical single cell models described in sections Electromechanical Single Cell Model to HFpEF Electromechanical Single Cell Model were pre-paced for a 1000 beats before being incorporated into the tissue model.

### Geometry and meshes

**Figure 1:**
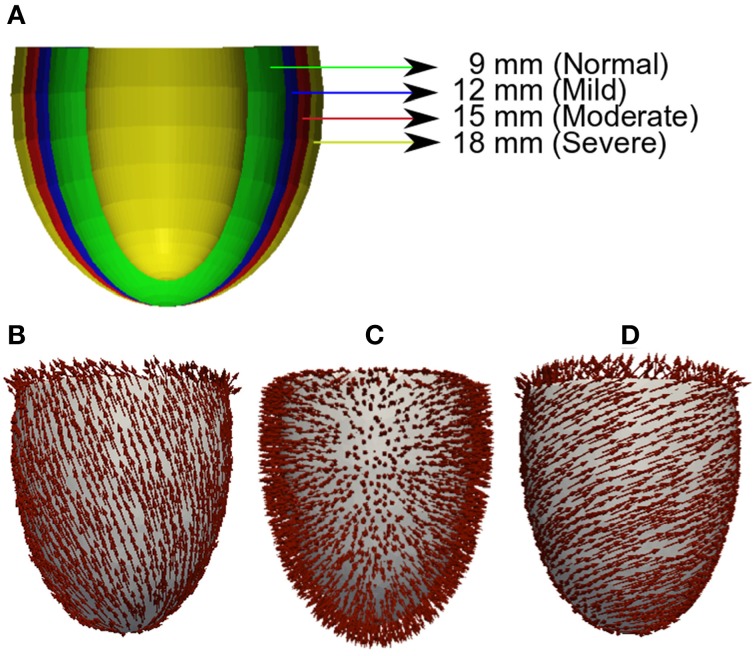
**Geometry and fiber directions in 3D left ventricle with varied wall thickness. (A)** Control and HFpEF concentric hypertrophic geometries—NORMAL (green, 9 mm), MILD (red, 12 mm), MODERATE (blue, 15 mm) and SEVERE (yellow, 18 mm). **(B)** Fiber direction. **(C)** Sheet direction. **(D)** Sheet-normal or cross-sheet direction.

For unbiased comparison between increasing severity of concentric LV hypertrophy in HFpEF, we used truncated ellipsoids for the 3D simulations (Figure 1A). Each LV geometry was segmented into distinct endocardial (60%), mid-myocardial (30%), and epicardial (10%) regions. The chosen cell proportion in each region reflects experimental data for cells spanning the left ventricular wall of the human heart (Drouin et al., 1995). There exists some controversy on the existence and functional role of MCELLs in the human heart (Wilson et al., 2011). However, we included MCELLs in our model on the basis of studies that give evidence of their existence in cells isolated from the right ventricle of patients with heart failure (Li et al., 1998) and in a perfused piece of left ventricular wall (“wedge preparation”) (Drouin et al., 1995). The conditional activation sites were projected from those of a healthy 34-year old male determined empirically across the ventricle wall and were validated by reproducing the activation sequence and QRS complex in the measured 64-channel ECG (Keller et al., 2009) of that person. Figures 1B–D shows the anisotropic fiber fields reconstructed by the algorithm in Rossi et al. (2014) for the fiber (Figure 1B), sheet (Figure 1C), and cross-sheet (Figure 1D) directions.

### Solving the electromechanics problem

The electromechanics problem consists of two sub-problems: the electrophysiology problem and the mechanics problem. The electrophysiology problem (Equation 14) was solved with a Strang splitting method (Sundnes et al., 2005) ensuring that the solution is second-order accurate. It was discretized in time using the Crank-Nicholson method (Burnett, 1987), which is also second-order accurate and discretized in space with Finite Elements (Ciarlet, 2002; Braess, 2007; Brenner and Scott, 2010). *I_ion_* in Equation (14) represents the single cell electromechanics model from which the active strain input to the 3D mechanics model for contraction is obtained. The system of ordinary differential equations (ODE) composing *I_ion_* was solved with a combination of the Rush-Larsen scheme (Rush and Larsen, 1978) and the CVODE solver (Cohen and Hindmarsh, 1996; Alan and Hindmarsh, 2005).

The mechanics problem (Equation 2) was also solved using the Finite element Method using the automated scientific computing library, FEniCS (Logg et al., 2012). The resulting nonlinear system of equations was solved iteratively using Newton's method to determine the equilibrium configuration of the system. Over a typical finite element domain, *P*_2_ elements (Braess, 2007; Brenner and Scott, 2010; Ern and Guermond, 2010; Boffi et al., 2013) were used to discretize the displacement variable *u*, while the pressure variable *p* was discretized with *P*_1_ elements (Braess, 2007; Brenner and Scott, 2010; Ern and Guermond, 2010; Boffi et al., 2013). This *P*_2_–*P*_1_ mixed finite element has been proven to ensure stability (Chamberland et al., 2010; Haga et al., 2012; Logg et al., 2012) and an optimal convergence rate (Hughes, 2000; Chamberland et al., 2010; Ern and Guermond, 2010).

The algorithm for solving the full electromechanics problem is as follows:

1. While time < tend:
  - Solve the electrophysiology problem for Δt_mechanics_ = 1 ms with active strain γ as output (Δt_electrophysiology_ = 0.01 ms).
  - Project γ from the electrophysiology mesh onto the mechanics mesh.
2. Solve the mechanics problem with γ as input.

## Results

### Model validation

**Figure 2:**
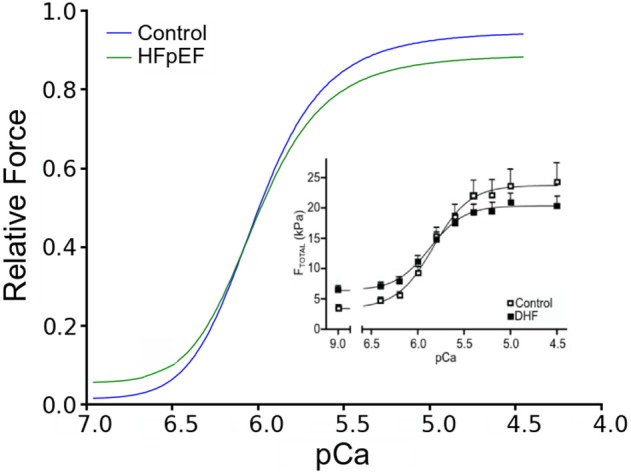
**Force-pCa relationship**. Simulated Force-pCa relation of control and HFpEF. Relative force is normalized to maximum value in control. Inset: Experimental force-pCa relation from human patients adopted from Borbély et al. (2005).

To validate the HFpEF model, we computed a steady-state force-calcium (F-pCa) relation for a sarcomere length (SL) of 2.2 μm for comparison with prior experimental data (Borbély et al., 2005). Results are shown in Figure 2. The model reproduced the differences in total and passive forces between control and HFpEF, which matched experimental data (inset).

### Functional consequences of HFpEF transmurally in the LV

**Figure 3:**
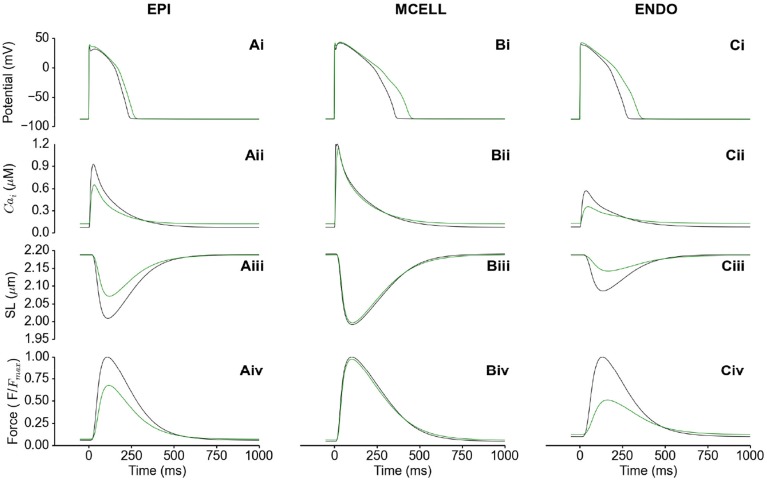
**Single cell simulations of HFpEF. (Ai–Ci)** Control (black) and HFpEF (green) action potentials in the EPI **(Ai)**, MCELL **(Bi)**, and ENDO **(Ci)** cell models. **(Aii–Cii)** Control (black) and HFpEF (green) cytosolic Ca^2+^ concentration in the EPI **(Aii)**, MCELL **(Bii)**, and ENDO **(Cii)** cell models. **(Aiii–Ciii)** Control (black) and HFpEF (green) sarcomere length (SL) in the EPI **(Aiii)**, MCELL **(Biii)**, and ENDO **(Ciii)** cell models. **(Aiv–Civ)** Control (black) and HFpEF (green) active force in the EPI **(Aiv)**, MCELL **(Biv)**, and ENDO **(Civ)** cell models. Values are normalized to Control maximum active force for each cell type.

Figure 3 shows the electromechanical consequences of HFpEF in a free-running cell from each of the epicardium (EPI) (Figures 3Ai–Aiv), mid-myocardium (MCELL) (Figures 3Bi–Biv) and endocardium (ENDO) (Figures 3Ci–Civ). HFpEF increased the action potential duration (APD_90_) in each of the EPI (Figure 3Bi), MCELL (Figure 3Bii) and ENDO (Figure 3Bi) cell types. APD_90_ was increased from 233 to 262 ms in EPI, 357 to 439 ms in MCELL and 274 to 348 ms in ENDO. These differences in APD_90_ across the ventricular wall are suggestive of possible T-wave changes and altered dispersion of repolarization in HFpEF. The cytosolic Ca^2+^ concentration [*Ca*^2+^]*_i_* was reduced in amplitude in all the cell types (Figures 3Aii–Cii), which led to a decrease in the SL shortening (Figures 3Aiii–Ciii) and consequently, a reduction in the active force (Figures 3Aiv–Civ). Of note is that the diastolic [*Ca*^2+^]*_i_* level was increased in HFpEF compared to control (Figures 3Aii–Cii) leading to incomplete relaxation (Figures 3Aiv–Civ). This electromechanical model predicted a combination of reduced contractile force and stress generation and incomplete relaxation as the pathophysiological mechanism of HFpEF (Zile et al., 2004; Borlaug and Paulus, 2010; Phan et al., 2012; Konstantinou et al., 2013; Liu et al., 2013; Zouein et al., 2013; Asrar Ul Haq et al., 2014).

### Functional consequences of HFpEF on ionic currents compared with HFrEF

**Figure 4:**
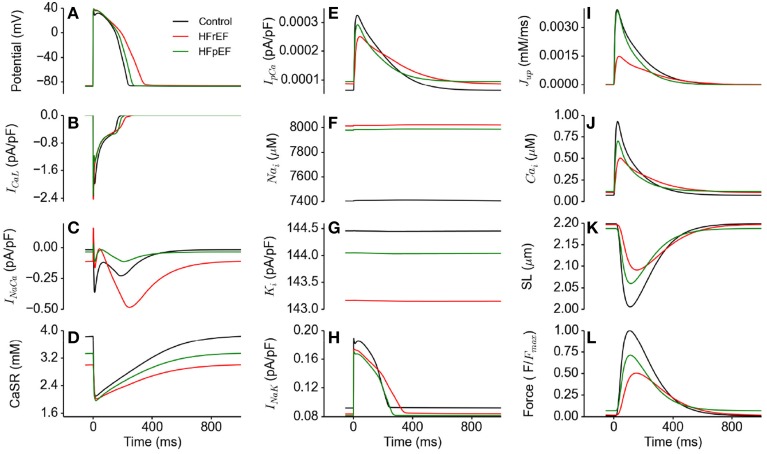
**Effects of HFpEF and HFrEF on underlying ion channel currents, concentrations and force generation. (A)** Control (black), HFpEF (green), and HFrEF (red) action potentials. **(B)** *I_CaL_* current profile in control (black), HFpEF (green), and HFrEF (red). **(C)** *I_NaCa_* current profile in control (black), HFpEF (green), and HFrEF (red). **(D)** SR Ca^2+^ content profile in control (black), HFpEF (green), and HFrEF (red). **(E)** *I_pCa_* current profile in control (black), HFpEF (green), and HFrEF (red). **(F)** [*Na*]_*i*_ time course in control (black), HFpEF (green), and HFrEF (red). **(G)** [*K*]*_i_* time course in control (black), HFpEF (green), and HFrEF (red). **(H)** *I_NaK_* current profile in control (black) HFpEF (green), and HFrEF (red). **(I)** *J_up_* (Ca^2+^ uptake via SERCA pump) profile in control (black), HFpEF (green), and HFrEF (red). **(J)** Ca^2+^ concentration in Control (black) HFpEF (green), and HFrEF (red) cytosolic. **(K)** Sarcomere length (SL) in control (black), HFpEF (green), and HFrEF (red). **(L)** Active force in control (black), HFpEF (green), and HFrEF (red). Values are normalized to Control maximum active force.

Figure 4 shows action potentials in control, HFrEF and HFpEF conditions (Figure 4A) computed from the EPI cell model, together with the corresponding ionic currents of *I_CaL_* (Figure 4B), *I_NaCa_* (Figure 4C), the SR content (Figure 4D), *I_pCa_* (Figure 4E), intracellular Na^+^ ([*Na*^+^]_*i*_; Figure 4F) and K^+^ ([*K*^+^]_*i*_; Figure 4G) concentrations and I_*Na*,*K*_. In comparison to the control condition, HFpEF and HFrEF increased the APD, which can be attributable to the increased inward Na^+^ current (I_*NaL*_) and decreased outward K^+^ current (I_*K*1_ and *I_NaK_*; see Table 1) (Figure 4H) (Glitsch, 2001; Workman et al., 2003; Bueno-Orovio et al., 2014). Accompanying these changes were increased [*Na*^+^]_*i*_ (Figure 4F) and reduced [*K*^+^]_*i*_ (Figure 4G). The difference in the amplitude of *I_CaL_* among control, HFpEF and HFrEF was small, but its duration was longer in HFpEF and longest in HFrEF (Figure 4B), possibly due to a secondary effect of a prolonged APD. In the HFpEF condition, a decreased *I_NaCa_* was observed, partially due to reduced Na^+^-Ca^2+^ exchanger (NCX) (see Table 1) and partially due to the reduction in the Na^+^-K^+^ pump activity as it indirectly regulated Ca^2+^ extrusion by the NCX (Barry et al., 1985; Bueno-Orovio et al., 2014). This resulted in a reduced sarcoplasmic reticulum (SR) content in HFpEF (Figure 4D), and consequently leading to a smaller SR Ca^2+^ release as demonstrated by the reduced amplitude of the SR content (Figure 4D). The activity of the sarcolemmal Ca^2+^ pump was increased during the diastolic period, but reduced in the systolic period (Figure 4E). These simulation results provided a cellular basis for the abnormal Ca^2+^ handling in HFpEF. Results from the MCELL and ENDO cell models showed similar behavior (these are shown in the Supplementary Material).

Unlike in HFpEF, there was an increased *I_NaCa_* (Figure 4C) due to the HFrEF model formulation involved a 175% NCX increase (Table 1). Figure 4C shows that NCX removed an excessive amount of Ca^2+^ from the cell in its forward mode compared to control, leading to a deficit in the SR Ca^2+^ content (Figure 4D), a greater reduction in [*Ca*^2+^]_*i*_ compared to HFpEF (Figure 4J) and consequently a lower active force (Figure 4L).

### Post-rest contraction properties of HFpEF

**Figure 5:**
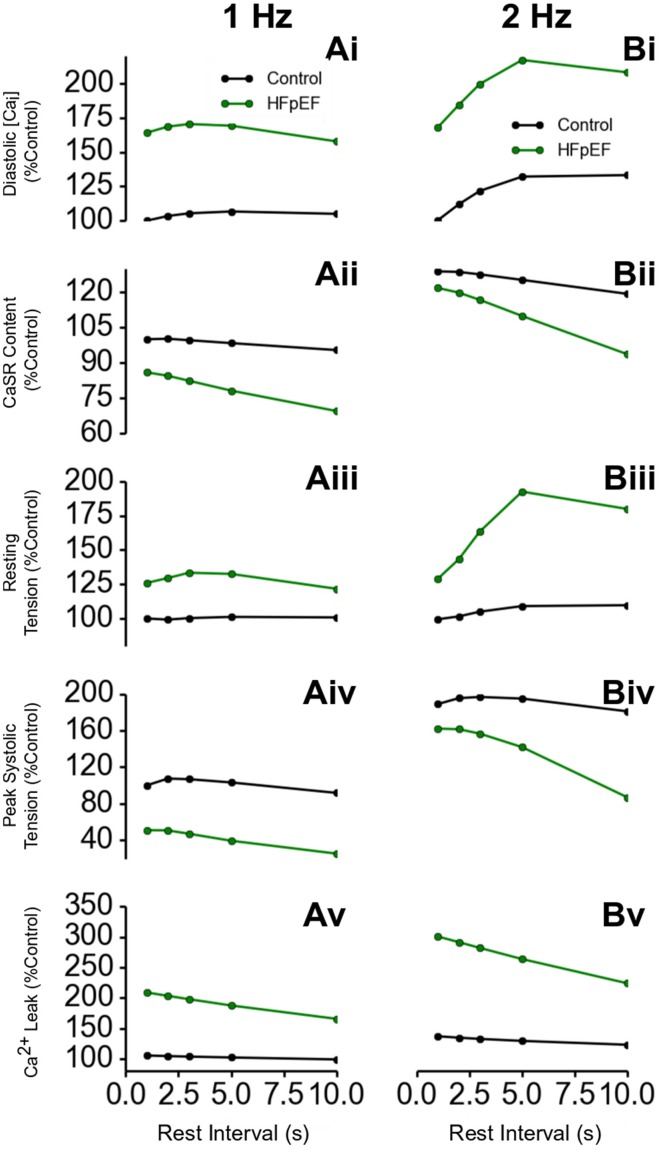
**Ca^2+^ handling and post-rest properties in HFpEF. (Ai,Bi)** Diastolic Ca^2+^ level in HFpEF relative to control at 1 Hz **(Ai)** and 2 Hz **(Bi)** pacing rates. **(Aii,Bii)** SR Ca^2+^ content level in HFpEF relative to control at 1 Hz **(Aii)** and 2 Hz **(Bii)** pacing rates. **(Aiii,Biii)** Resting tension in HFpEF relative to control at 1 Hz **(Aiii)** and 2 Hz **(Biii)** pacing rates. **(Aiv,Biv)** Peak systolic tension in HFpEF relative to control at 1 Hz **(Aiv)** and 2 Hz **(Biv)** pacing rates. **(Av,Bv)** SR Ca^2+^ Ca leak in HFpEF relative to control at 1 Hz **(Av)** and 2 Hz **(Bv)** pacing rates.

Further simulations were performed to investigate the rate-dependent impact of HEpEF on Ca^2+^ handling. Results are shown in Figure 5, which shows the outcome of the PRC protocol at a pacing rate of 1 Hz (Figures 5Ai–Av) and 2 Hz (Figures 5Bi–Bv). Diastolic [*Ca*^2+^]_*i*_ level in HFpEF increased by ~75% relative to control at all resting intervals (Figures 5Ai,Bi). This led to an increase in resting tension (Figures 5Aiii,Biii). Though there was a significant increase in diastolic *Ca*^2+^]_*i*_ level, the SR content was lower in HFpEF than in control (Figures 5Aii,Bii) as was peak systolic tension (Figures 5Aiv,Biv). This is counter-intuitive as with an increased diastolic concentration of Ca^2+^ in the cytosol, one would expect greater Ca^2+^ sequestration into the SR, and therefore an increased Ca^2+^ content leading to an increased Ca^2+^ release from the SR, resulting in a greater Ca^2+^ transient amplitude and a greater systolic tension. The reduction in the peak systolic Ca^2+^ level and the corresponding tension and inefficient SR Ca^2+^ activity was due to excessive leak of Ca^2+^ from the SR (Figures 5Av,Bv). These results were more pronounced at a pacing rate of 2 Hz (Figures 5Bi–Bv) because of the shorter duration between beats allowing less recovery time for Ca^2+^ cycling processes.

### Sensitivity of diastolic relaxation to NCX

**Figure 6:**
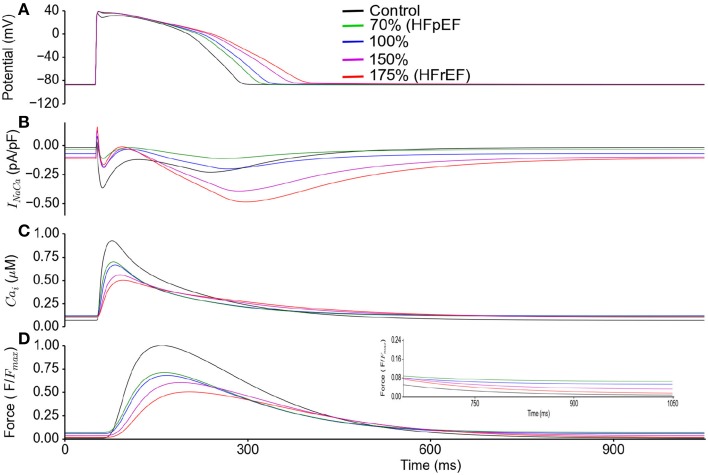
**HFpEF Model sensitivity to increasing NCX activity and its influence on incomplete relaxation in the cellular model**. Simulation results were compared between control (in absence of ionic current remodeling) and HFpEF condition. HFpEF simulations were performed with parameters as listed in Table 1, but with NCX activity changing from 70% (HFpEF condition) to 100, 150, and 175% (HFrEF condition) of the control value. **(A)** Action potential **(B)** *I_NaCa_* current profile. **(C)** Ca^2+^ concentration. **(D)** Active force. Values are normalized to maximum active force in control. (Inset: magnified diastolic phase).

In Figure 6, effects of a systematic change of NCX in HFpEF model from 70% (HEpEF) to 175% (HFrEF) on the action potentials (Figure 6A), *I_NaCa_* (Figure 6B), [*Ca*^2+^]*_i_* (Figure 6C) and active force (Figure 6D) are shown. Diastolic relaxation was impaired when NCX was at 70% of the control value (e.g., for HFpEF), but gradually improved with increasing NCX activity to 100 and 150% of the control value. It became normal at 175% (e.g., for HFrEF; Figure 6D) of the control value. Peak relative force during systole occurred later and with lower amplitude with increasing NCX activity.

### 3D electromechanical consequences of HFpEF in hypertrophic geometries

**Figure 7:**
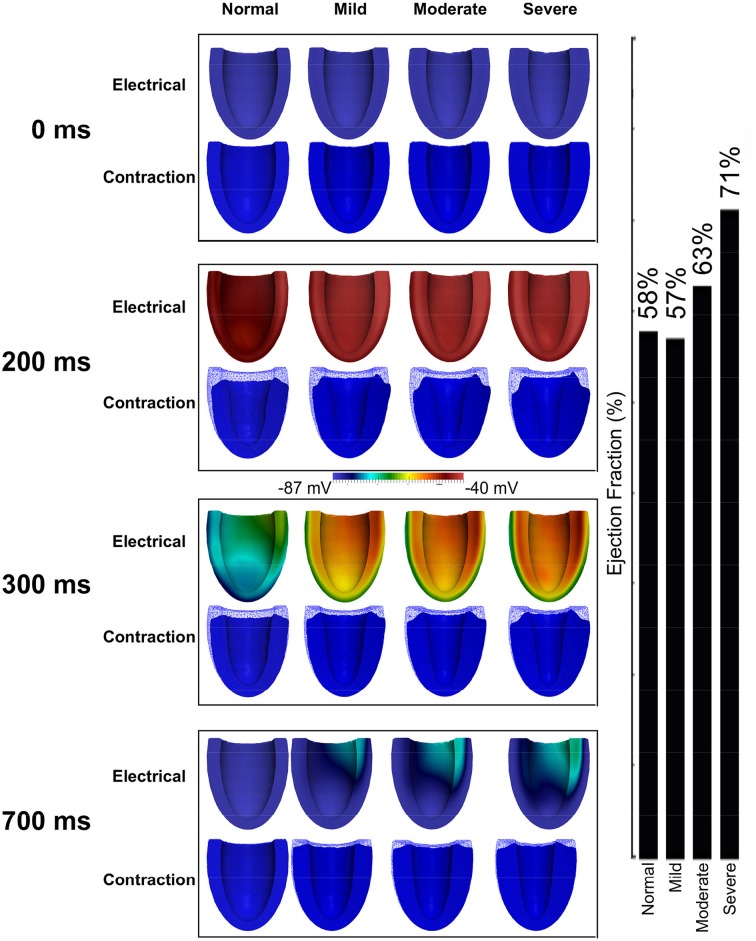
**Effects of HFpEF on 3D electro-mechanics**. Electrical wave propagation and mechanical contraction at 0, 200, 300, and 700 ms in NORMAL, MILD, MODERATE, and SEVERE HFpEF hypertrophic cases. (Far right) Ejection fraction in NORMAL, MILD, MODERATE, and SEVERE HFpEF hypertrophic cases.

Figure 7 shows the results of incorporating the cellular HFpEF electromechanical models into three-dimensional truncated ellipsoid representations of the LV. Effects of varying degrees of left ventricular hypertrophy on the ejection fraction are also shown. The LV was at rest before activation (0 ms). At about 200 ms, the LV was completely activated and contraction was underway in the NORMAL, MILD, MODERATE, and SEVERE conditions of hypertrophy. By 300 ms, repolarization had commenced and the LV in all conditions was undergoing relaxation. At 700 ms, repolarization was completed in all the conditions; however, relaxation was still on going in the hypertrophic cases but was complete in the NORMAL condition. The LVEF in NORMAL, MILD, MODERATE, and SEVERE was 58, 57, 63, and 71% respectively showing that LVEF was increased with increasing the end-diastolic wall thickness. These simulation results can account for incomplete relaxation and preserved LVEF at the 3D organ level, whilst at the cellular level, the activation force and cell sarcolemmal shortening are dramatically impaired in the HFpEF condition. This is attributable to the hypertrophied ventricle wall.

### Stress distribution in HFpEF

**Figure 8:**
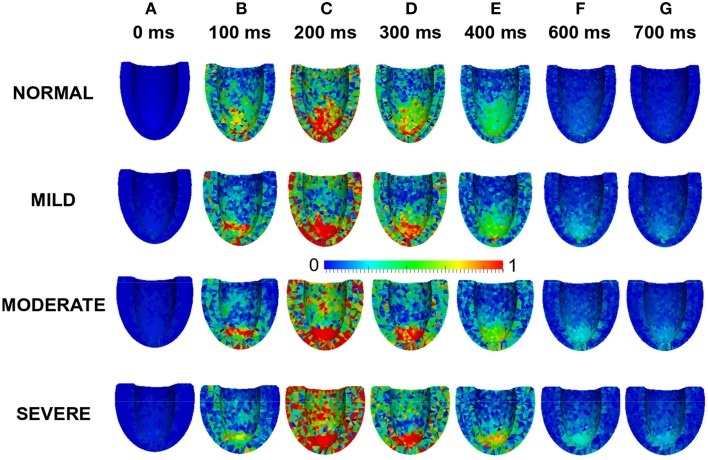
**Effects of HFpEF on stress magnitude distribution**. Stress magnitude distribution at 0 ms **(A)**, 100 ms **(B)**, 200 ms **(C)**, 300 ms **(D)**, 400 ms **(E)** 600 ms **(F)**, and 700 ms **(G)** in NORMAL, MILD, MODERATE, and SEVERE hypertrophic HFpEF cases.

Effects of LV wall thickness on the spatial distribution of stress was investigated. Figure 8 shows a 4-chamber view of the magnitude of the stress distribution across the LV in control and varied hypertrophic conditions (Figures 8A–G). At 0 ms, the stress magnitude across the ventricle was low in all the conditions as excitation had yet to commence (Figure 8A). By 100 ms, when the LV was electrically activated, there was developed stress in all cases with the greatest stress in apex of the MODERATE LV (Figure 8B). At 200 ms (Figure 8C), there was considerable stress in the LV in all conditions with the greatest stress intensity in SEVERE. The hypertrophic LV also had greater stress intensity in the LV apex and epicardium compared to NORMAL. The situation was similar at 300 ms (Figure 8D) and 400 ms (Figure 8E) except that there was progressive relaxation and the stress intensity was less than at 200 ms. By 600 ms (Figure 8F), stress in most of the NORMAL LV had reduced considerably while the hypertrophic LVs still showed ~25% stress. At 700 ms, stress in the NORMAL LV was negligible but still about ~20% in the hypertrophic cases. These simulation results showed that increased wall thickness led to increased tissue stress though, at the cellular level, the active force was reduced during the time course of action potentials in HEpEF condition.

### Strain distribution in HFpEF

**Figure 9:**
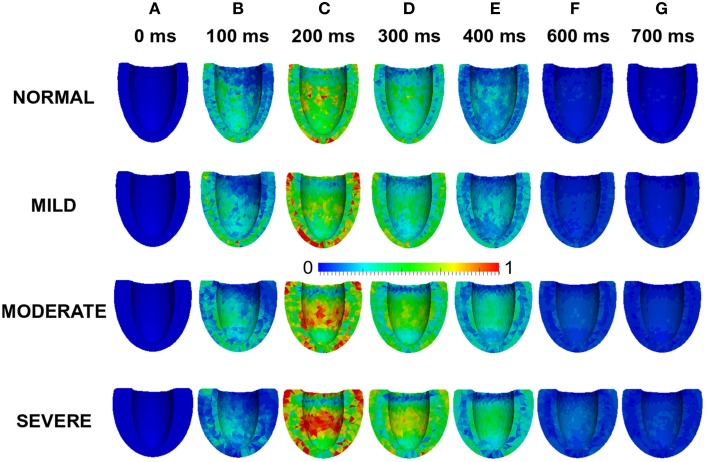
**Effects of HFpEF on strain magnitude distribution**. Strain magnitude distribution at 0 ms **(A)**, 100 ms **(B)**, 200 ms **(C)**, 300 ms **(D)**, 400 ms **(E)** 600 ms **(F)**, and 700 ms **(G)** in NORMAL, MILD, MODERATE, and SEVERE hypertrophic HFpEF cases.

Effects of wall thickness on the spatial distribution of strain was also analyzed. Figure 9 also shows a 4-chamber view of the magnitude of the strain distribution across the LV in control and hypertrophic conditions during the time course cardiac excitation (Figures 9A–G). Strain developed from the rest state (Figure 9A) to ~50% at 100 ms (Figure 9B) in all cases with the smallest strain magnitude in SEVERE. By 200 ms (Figure 9C), strain magnitude had reached a distribution of between 50 and 100% in all cases with SEVERE having the highest strain intensity. This was concentrated in the epicardial to mid-wall regions and the middle segment of the LV. The situation was similar at 300 ms (Figure 9D) and 400 ms (Figure 9E) except that the strain intensity was reduced as relaxation was underway. The strain magnitude reduced further at 600 ms (Figure 9F) to ~10–15% in NORMAL, ~25% in MILD, ~25–30% in MODERATE and ~30–35% in SEVERE. The strain was mainly concentrated in the LV apex in all cases. By 700 ms (Figure 9G), strain magnitude was ~5% in NORMAL, 10–25% in all the hypertrophic cases. These simulation results showed an increased residual of strain in cardiac tissue due to elevated diastolic Ca^2+^ level, which was consistent with observed incomplete relaxation of the LV in HEpEF condition.

## Discussion

### Summary of major findings

In this study we have developed, for the first time, a family of multilevel models for the electro-mechanics of the left ventricle in the setting of HFpEF, at the cellular and 3D organ levels. These models incorporated detailed HFpEF-related ion channel remodeling and impaired Ca^2+^ homeostasis at the cellular level, and concentric hypertrophy of the left ventricle wall at the organ level. Our major findings are: (i) with impaired Ca^2+^ handling and ion channel remodeling in HFpEF, the action potential duration of ventricular cells are prolonged, together with an elevated diastolic Ca^2+^ concentration, but a decreased systolic Ca^2+^ level. Such an elevated diastolic Ca^2+^ concentration provides a cellular basis for incomplete ventricular relaxation at the organ level; (ii) at the cellular level the active force and sarcomere length shortening is reduced during the time course of action potentials in HFpEF. However, at the organ level, tissue stress and strain is increased due to the increased wall thickness of concentric LV hypertrophy; (iii) the impaired Ca^2+^ homeostasis becomes more pronounced at high stimulation rates; and (iv) reduction of *I_NaCa_* in the HEpEF model is the most influential factor on impaired relaxation dynamics. Collectively, these simulations predict the key features of HFpEF observed clinically, and also provide insights for understanding the cellular and tissue bases of impaired electro-mechanics of the heart in HFpEF.

### Cellular basis of impaired cardiac electro-mechanics

The results of our study suggest that at the cellular level the observed impaired cardiac electro-mechanics (such as reduced cell length shortening and active force) are attributable to alterations in cellular Ca^2+^ homeostasis and action potentials. In simulations, we observed an elevated diastolic intracellular Ca^2+^ concentration, but a reduced systolic Ca^2+^ concentration. The elevated diastolic Ca^2+^ concentration is due to an increased Ca^2+^ leak from the SR (see Table 1), and the reduced systolic Ca^2+^ concentration can be explained by a reduced Ca^2+^ release from the SR as a consequence of a decreased SR content (Figure 4D). The observed APD prolongation is attributable to the augmented late-Na^+^ current, reduced potassium channel currents and *I_NaK_* as seen in the heart failure condition (see Table 1). These results were more pronounced at a pacing rate of 2 Hz (Figures 5Bi–Bv) because of the shorter duration between beats allowing less recovery time for Ca^2+^ cycling processes. This is related to changes in mechanical and relaxation restitution, which correlate physiologically to the recovery kinetics of Ca^2+^ release mechanisms and sequestration capacity of the SR (Franz et al., 1983; Burkhoff et al., 1984; Prabhu and Freeman, 1995; Zaugg et al., 1995; Kjørstad et al., 2007).

In simulations, we also observed an increased intracellular Na^+^ concentration and a reduced intracellular K^+^ concentration (Figures 4F,G), which may be attributable to the augmented *I*_NaL_ and reduced *I_NaK_* (see Table 1). The altered Na^+^ and K^+^ homeostasis may also impair Ca^2+^ homeostasis. During the time course of an action potential, there was a reduced *I_NaK_*, which also contributed partially to an increase of [*N*a^+^]_*i*_ and a decrease in [*K*^+^]_*i*_ within the cytosol. NCX extrudes Ca^2+^ from the cytoplasm and imports Na^+^ into it. However, with the build-up of Na^+^ in the cytoplasm as a result of the reduced activity of the Na^+^-K^+^ pump, the forward mode activity of NCX was reduced, but its reverse mode activity was enhanced. This led to an increase in Na^+^ extrusion coupled to Ca^2+^ import (see Figure 4C) leading to an increased Ca^2+^ concentration at diastolic phase but a reduced Ca^2+^ concentration at systolic phase. Consequently, this led to a decreased cell length shortening and active force in the systolic phase.

In addition, our simulation results showed that the SR Ca^2+^ content was reduced compared to control condition, though the Ca^2+^ release from the SR was compromised. This is attributable to an excessive Ca^2+^ leak from the SR. It was shown that SR Ca^2+^ leak was ~100% greater in HFpEF as compared to control (Figure 5). The excessive Ca^2+^ leak from the SR and the reduced activity of NCX produced an increased diastolic Ca^2+^ level, leading to incomplete relaxation and an increase in the active resting tone. This implies that complete relaxation will never be achieved regardless of the duration of diastole.

The simulation results discussed above were based on our HFpEF model developed from work by Zile and Gaasch (2011) and Selby et al. (2011). To our knowledge these are the only two studies that provide any notion of electrophysiological changes in HFpEF relative to control conditions. As the only difference between the HFpEF and the HFrEF models is a 30% NCX reduction in the former compared to control and a 175% increase in the latter (Table 1), we investigated whether changes in NCX activity could convert HFpEF to HFrEF and vice-versa, and therefore affect myocyte relaxation dynamics (Figure 6). This indeed proved to be the case. The results imply that a change in NCX activity is the dominant factor leading to impaired diastolic relaxation in HFpEF.

### Effects of concentric hypertrophy of the ventricle wall on cardiac electro-mechanics

Increased ventricular wall thickness has dramatic impacts on cardiac electro-mechanics. This is illustrated by the effects of varied degrees of hypertrophy on the ejection fraction in HFpEF condition (Figure 9). In simulations, the wall thickness of the LV varied from 9 mm (normal) to 12, 15, and 18 mm, mimicking varying degrees of hypertrophy. Though at the cellular level, cell length shortening and active force was decreased in HFpEF condition, the LVEF was preserved and increased with the increase of LV wall thickness. This agrees with what is seen clinically (Borlaug and Paulus, 2010; Phan et al., 2012; Liu et al., 2013; Zouein et al., 2013) and with previous modeling data (MacIver and Townsend, 2008; MacIver, 2010b, 2011). Our simulations confirm that the preserved LVEF in HFpEF is due to the thicker wall of the ventricle arising from concentric hypertrophy.

Taken together, our simulations suggest a possible pathway and mechanism underlying cardiac dysfunction in HFpEF. Any co-morbidities such as diabetes, hypertension, inflammation and/or hypertrophy may cause the ion channel and myofilament remodeling in myocardial cells. Cellular remodeling results in abnormal Ca^2+^ homeostasis, which in turn lead to abnormalities of contraction and incomplete relaxation of the LV with a persistent active resting tone. These abnormalities combine to increase the length of time to which the myocardium is subjected to stress prolonging systole and resulting in abnormal energy utilization and less efficient ejection of the stroke volume.

### Relevance to previous studies

Lacombe et al. (2007) investigated the underlying mechanisms of diastolic dysfunction in type 1 diabetic rats. They observed no significant change in *I*_CaL_, a reduction in Ca^2+^ transient amplitude and prolongation in its decay, a reduction in SR Ca^2+^ load and a decrease in the expression of sarco(endo)plasmic reticulum Ca^2+^-ATPase-2a (SERCa) protein levels. They concluded that impairment of Ca^2+^ reuptake during myocyte relaxation contributed to diastolic dysfunction, with preserved global systolic function.

Selby et al. (2011) carried out a study to evaluate tachycardia-induced relaxation abnormalities in myocardium from patients with a normal ejection fraction. They observed incomplete relaxation with increased diastolic tension development at increasing pacing rates, significant resting tone and disproportionately elevated Ca^2+^ loads due to reduced sarcolemmal Ca^2+^ extrusion reserve. However, their patients did not carry a clinical diagnosis of heart failure.

MacIver and Townsend (2008) performed a mathematical study on HFpEF to determine the effect of changes in LV hypertrophy on stroke volume and LVEF. They concluded from their model that the preserved LVEF in HFpEF patients was a result of LV hypertrophy, which amplified absolute radial wall thickening in the setting of reduced long-axis shortening. MacIver also showed, using a simple abstract model, that remodeling was necessary to normalize stroke volume and suggested that regulation of end-diastolic volume was a primary compensatory mechanism in heart failure (MacIver, 2010b). However, their models did not consider the contribution of cardiomyocytes, coupled electrical wave propagation or nonlinear anisotropic cardiac mechanics.

Our simulation results are in agreement with and extend the findings of these previous studies (Lacombe et al., 2007; MacIver and Townsend, 2008; Selby et al., 2011), adding new evidence that impaired Ca^2+^ homeostasis (such as reduced systolic Ca^2+^ concentration and elevated diastolic Ca^2+^ concentration) together with hypertrophied wall underlie the key features of HFpEF with preserved ejection fraction and incomplete end-diastolic relaxation.

### Limitations

In addition to acknowledged limitations of both the ORd electrophysiology model (O'Hara et al., 2011) and the (Tran et al., 2010) myofilament model, as experimental data show a reduction in NCX activity in diastolic dysfunction compared to control (Zile and Gaasch, 2011), we made a 30% reduction in NCX activity in our HFpEF model as there was no quantitative data available at the time of this study. In HFpEF patients, collagen production results in interstitial fibrosis (Heymans et al., 2005; Konstantinou et al., 2013), which we considered by reducing the intracellular electrical conductivities by 20% due to a lack of quantitative data. The HFpEF model also relaxes somewhat faster than the experimental data, which would imply that the effects of incomplete relaxation would be even greater than we have shown. As there are no data available, we assumed the same degree of ion channel remodeling for the hypertrophied ventricles and also assumed the same distribution of ENDO, MCELL, and EPI cell types across the ventricular wall. We also did not consider electromechanical feedback between the electrical wave propagation and mechanically contracting ventricles. Finally, the use of a fluid-structure interaction model with the interaction of blood and the myocardial wall to determine pressure boundary conditions would allow a more realistic pressure profile. Whilst it is important that these potential limitations are stated, they do not fundamentally alter the principal conclusions of this study.

## Conclusion

We have developed a novel, biophysically detailed model of HFpEF and used it to investigate the cellular mechanisms underlying myocardial Ca^2+^ homeostasis in HFpEF. We observed an elevated diastolic [*Ca*^2+^]*_i_* level, a reduction in SR Ca^2+^ content and reduced SR Ca^2+^ release, a reduction in SR Ca^2+^ sequestration, an increase in resting tension, incomplete relaxation, reduced systolic stress and prolonged stress and strain durations. These mechanisms suggest that in HFpEF patients, impaired Ca^2+^ handling principally caused by reduction in NCX activity is a dominant abnormality in the condition that explains the mechanisms for impaired cardiac electro-mechanics in HFpEF.

### Conflict of interest statement

The authors declare that the research was conducted in the absence of any commercial or financial relationships that could be construed as a potential conflict of interest.
