# Supplementary material for: Abnormal calcium homeostasis in heart failure with preserved ejection fraction is related to both reduced contractile function and incomplete relaxation: an electromechanically detailed biophysical modeling study
Source: Front Physiol. 2015 Mar 20;6:78. doi: 10.3389/fphys.2015.00078 (PMC4367530; doi:10.3389/fphys.2015.00078)
Supplement: Supplementary file 1 [file Presentation1.PDF]

Appendix

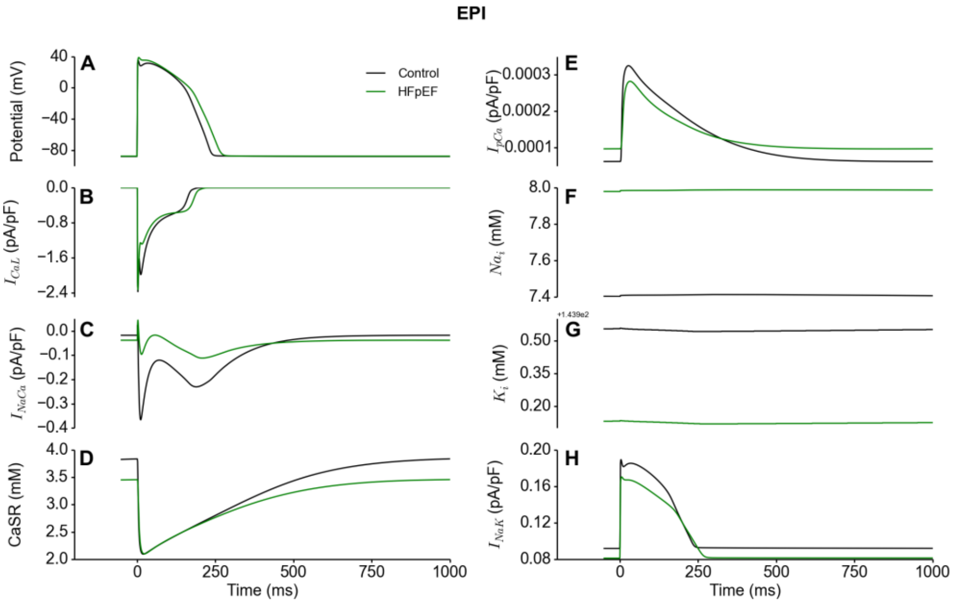

Figure S1. Effects of HFpEF on underlying ion channel currents and ionic homeostasis from EPI cell. (A) Control (black) and HFpEF (green) action potentials. **(B)** I_{CaL} current profile in control (black) and HFpEF (green). **(C)** I_{NaCa} current profile in control (black) and HFpEF (green). **(D)** SR Ca^{2+} content profile in control (black) and HFpEF (green). **(E)** I_{pCa} current profile in control (black) and HFpEF (green). **(F)** $[Na]_i$ time course in control (black) and HFpEF (green). **(G)** $[K]_i$ time course in control (black) and HFpEF (green). **(H)** I_{NaK} current profile in control (black) and HFpEF (green).

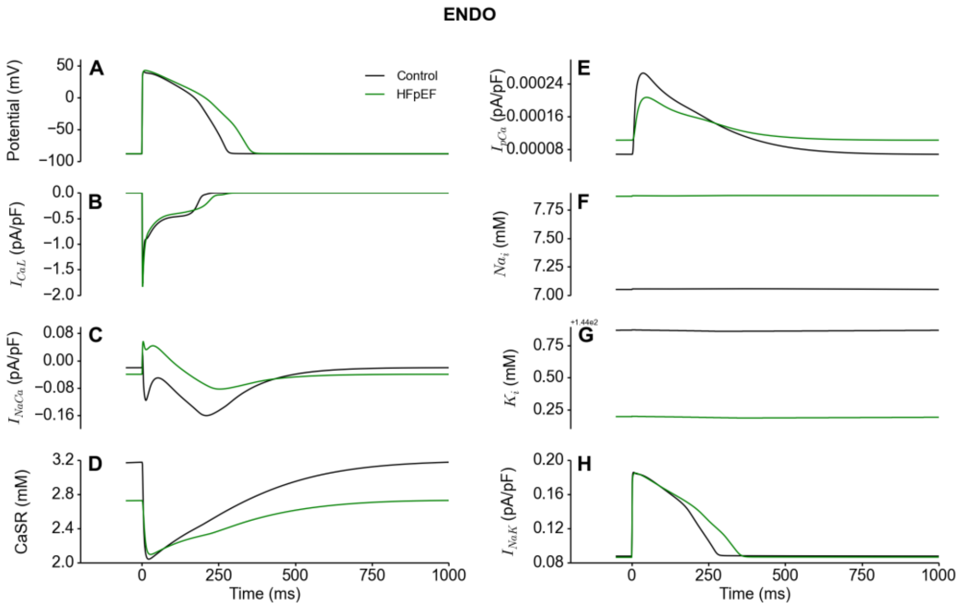

Figure S2. Effects of HFpEF on underlying ion channel currents and ionic homeostasis from ENDO cell. (A) Control (black) and HFpEF (green) action potentials. **(B)** I_{CaL} current profile in control (black) and HFpEF (green). **(C)** I_{NaCa} current profile in control (black) and HFpEF (green). **(D)** SR Ca^{2+} content profile in control (black) and HFpEF (green). **(E)** I_{pCa} current profile in control (black) and HFpEF (green). **(F)** $[Na]_i$ time course in control (black) and HFpEF (green). **(G)** $[K]_i$ time course in control (black) and HFpEF (green). **(H)** I_{NaK} current profile in control (black) and HFpEF (green).

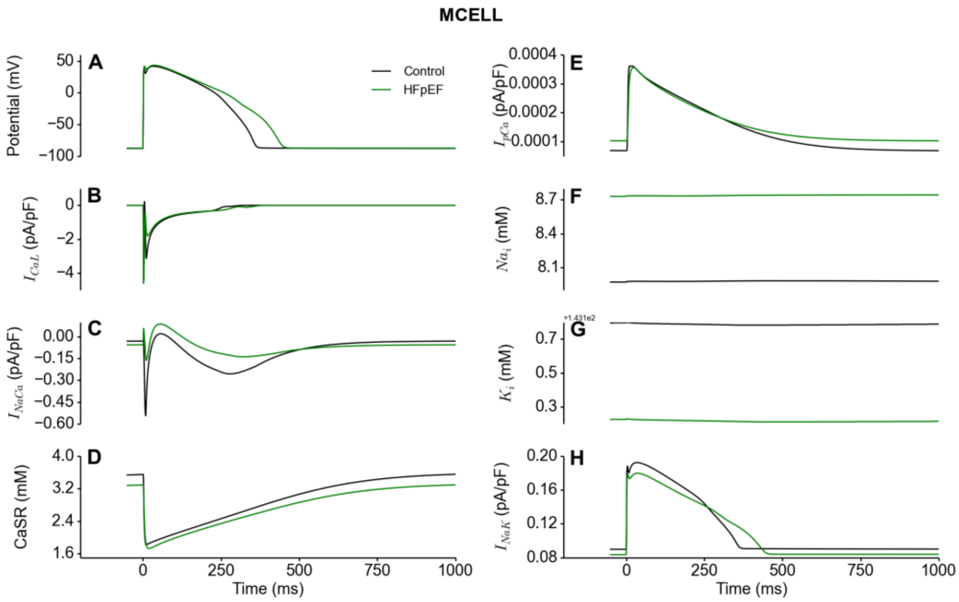

Figure S3. Effects of HFpEF on underlying ion channel currents and ionic homeostasis from M-cell. (A) Control (black) and HFpEF (green) action potentials. **(B)** I_{CaL} current profile in control (black) and HFpEF (green). **(C)** I_{NaCa} current profile in control (black) and HFpEF (green). **(D)** SR Ca^{2+} content profile in control (black) and HFpEF (green). **(E)** I_{pCa} current profile in control (black) and HFpEF (green). **(F)** $[Na]_i$ time course in control (black) and HFpEF (green). **(G)** $[K]_i$ time course in control (black) and HFpEF (green). **(H)** I_{NaK} current profile in control (black) and HFpEF (green).
